# Two-Stage Prediction of the Effects of Imidazolium and Pyridinium Ionic Liquid Mixtures on Luciferase

**DOI:** 10.3390/molecules19056877

**Published:** 2014-05-23

**Authors:** Hui-Lin Ge, Shu-Shen Liu, Bing-Xia Su, Xiang-Wei Zhu

**Affiliations:** 1 Hainan Provincial Key Laboratory of Quality and Safety for Tropical Fruits and Vegetables, Analysis and Testing Center, Chinese Academy of Tropical Agricultural Sciences, Haikou 571101, China; E-Mail: sublin22@163.com; 2 Key Laboratory of Yangtze Aquatic Environment, Ministry of Education, College of Environmental Science and Engineering, Tongji University, Shanghai 200092, China; 3 College of Resource and Environment, Qingdao Agricultural University, Qingdao 266109, China; E-Mail: shinevip@163.com

**Keywords:** ionic liquids, luciferase, molecular level, joint toxicity, concentration addition, independent action, two-stage prediction, NOEC, mixture risk assessment

## Abstract

The predicted toxicity of mixtures of imidazolium and pyridinium ionic liquids (ILs) in the ratios of their EC_50_, EC_10_, and NOEC (no observed effect concentration) were compared to the observed toxicity of these mixtures on luciferase. The toxicities of EC_50_ ratio mixture can be effectively predicted by two-stage prediction (TSP) method, but were overestimated by the concentration addition (CA) model and underestimated by the independent action (IA) model. The toxicities of EC_10_ ratio mixtures can be basically predicted by TSP and CA, but were underestimated by IA. The toxicities of NOEC ratio mixtures can be predicted by TSP and CA in a certain concentration range, but were underestimated by IA. Our results support the use of TSP as a default approach for predicting the combined effect of different types of ILs at the molecular level. In addition, mixtures of ILs mixed at NOEC and EC_10_ could cause significant effects of 64.1% and 97.7%, respectively. Therefore, we should pay high attention to the combined effects in mixture risk assessment.

## 1. Introduction

Organisms are usually exposed to pollutant mixtures in the environment [1]. The assessment and prediction of mixture effects is generally based on two additive reference models: concentration addition (CA) and independent action (IA). CA is generally applied in predicting the mixture toxicity of similarly acting chemicals [2], while IA is generally appropriate for the mixture of dissimilarly acting chemicals [3]. Later, Junghans proposed a two-stage prediction (TSP) method that combined CA and IA in a stepwise manner [4]. TSP has been used to predict mixture toxicities of different types of chemicals [5,6].

Ionic liquids (ILs) are novel organic salts with low melting points that have enormous potential for industrial use as green replacements for harmful volatile organic solvents [7]. The application areas of ILs include catalysis, extraction, synthesis, dissolution, nuclear industry, and food science [8]. Although ILs will not cause air pollution because of their negligible vapor pressures, some of them still present a non-negligible solubility in water, thus leading to aquatic environmental risks [9]. Several studies have reported the biological effects of single ILs on the basis of different toxicological test systems such as enzymes (e.g., acetylcholinesterase), bacteria (e.g., *Vibrio fischeri*), algae (e.g., *Selenastrum capricornutum*), mammalian cells (e.g., MCF-7 cell), plants (e.g., wheat and cress), invertebrates (e.g., zebra mussel), and vertebrates (e.g., *Danio rerio*) [10,11,12,13,14,15,16].

Mixtures of ionic liquids are increasingly applied in practical applications such as solvents for chemical synthesis and process chemistry, electrochemistry, chromatography and heat transfer fluids [17]. It is of great importance to predict and assess their combined effects before any likely industrial release into the environment. Several studies have reported the combined effects of ILs. For example, the effects of imidazolium IL mixtures on *Triticum aestivum* and *Scenedesmus vacuolatus* were underestimated by the CA and IA models [18]. Imidazolium ILs can cause multiple toxic interactions (addition, synergism, and antagonism) in different composition and concentration ranges [19]. The synergism and antagonism of imidazolium ILs are well correlated to the concentration ratio of ILs with BF_4_^−^[20]. Nevertheless, little is known about the combined toxicity of different types of ILs, including imidazolium and pyridimium ILs. Important questions are: (i) can their mixture toxicities be predicted by CA, IA or TSP; (ii) what is the type of their toxic interaction (addition, synergism or antagonism); and (iii) what is the location relationship among the concentration–response curves (CRCs) predicted by CA, IA, and TSP for their mixtures? Although some of these issues have been preliminarily investigated in single types of ILs such as imidazolium ILs, it is necessary to further explore and validate this for mixtures of different types of ILs.

Luciferase luminescence is a process in which the luciferase catalyzes the oxidation of the substrate D-luciferin and the energy transfer from ATP to D-luciferin to yield light. Because the luciferase luminescence can be affected by chemicals, it has been applied as a test system to characterize the toxicities of single chemicals [21] and mixtures [22]. Based on the luciferase toxicity test, we demonstrated that the effects of mixtures of ten ILs with J-shaped CRC can be predicted by the CA model [23]. In the present study, we evaluate the effects of mixtures of imidazolium and pyridimium ILs with S-shaped CRC. Mixture effects of ILs in the ratios of their EC_50_, EC_10_, and NOEC on luciferase were tested based on microplate toxicity analysis. Combined effects were evaluated by comparing the predicted effects by CA, IA, and TSP to the observed effects of these mixtures.

## 2. Results and Discussion

### 2.1. Single Toxicity

**Figure 1:**
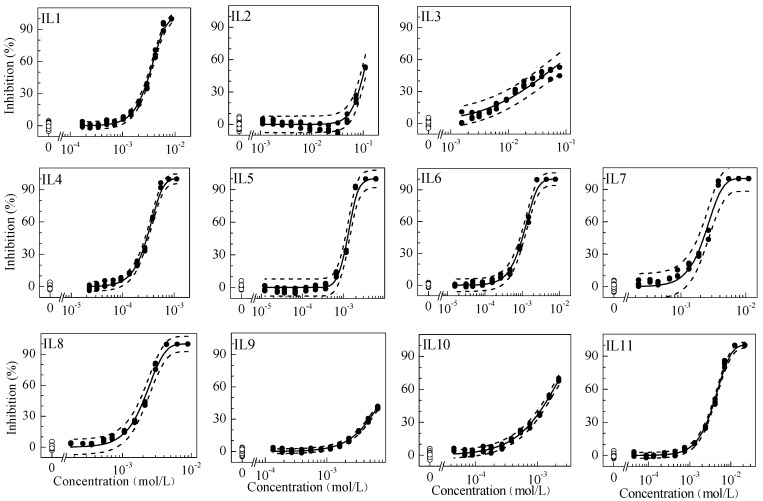
Concentration–response curves for the inhibitory effects of individual ionic liquids on luciferase luminescence. Hollow circle: blank control; solid circle: data observed; solid line: model fit; dash line: confidence interval.

The regression models and the estimated parameters of the toxicity of single ILs on luciferase are summarized in Table 1 and the resulting CRCs are visualized in Figure 1. These CRCs can be effectively described by the Logit or Weibull model with RMSE (<0.06) and *R*^2^ (>0.95), indicating good relationships between the exposure concentrations of ILs and the inhibition effects. The variability of the blank control in our test was controlled within ±10%. All EC_50_, EC_10_ and the NOEC values are shown in Table 1. According to their EC_50_s, the toxicity order of single ILs was IL4 > IL6 > IL5 > IL10 > IL8 > IL7 > IL1 > IL11 > IL9 > IL3 > IL2 (for the meaning of the abbreviations see below). Both the present study and a previous study [23] all indicated that the ILs with BF_4_^−^ anions showed higher luciferase toxicity. Moreover, ILs with BF_4_^−^ anions were also inclined to produce higher toxicity on other organisms such as the luminescent bacterium Q67 [24] and MCF-7 mammalian cells [13]. The high toxicity of ILs with fluoride-containing anions could be due to the hydrolytic cleavage resulting in the formation of free fluoride ions [25]. Egorova and Ananikov have reviewed the main factors modulating the toxicity of ILs: (i) alkyl chain length and side chain functionalization in the cation; (ii) nature of the anion and cation; (iii) mutual influence of the anion and cation [26].

By inserting the NOEC values (Table 1) into the concentration-response functions (*F*), it can be calculated that they correspond to effects ranging from 0.1% for IL5 to 11.3% for IL3 with an arithmetic mean of 3%. The NOEC was obtained based on conventional hypothesis testing for each tested concentration. Many ecotoxicologists are aware of the shortcomings of the NOEC. The most often cited concerns include: the NOEC is constrained to be one of the test concentrations; confidence intervals cannot be calculated for NOEC; NOEC cannot always be determined; NOEC is controlled by the concentration interval, the data variability, the number of replicates, and the selected significance level [27]. On the other hand, the EC*_x_* (the concentration causing an effect of *x* percent) was estimated from a concentration-response model based on statistical regression analysis of whole data sets, so NOECs are innately more variable than EC*_x_* point estimates. Low effect EC*_x_* values have been suggested as appropriate alternatives to NOEC [28]. Using EC*_x_* to replace NOEC requires the value of *x* to be specified. Van der Hoeven *et al*. suggested that the preferred value of *x* would be 5 or 10 percent [29]. In fact, the confidence interval for low effect EC*_x_* may be very wide and sometimes even missing such as the EC_10_ of IL3 and IL7 in our study, so EC_10_ may be a more appropriate choice to replace NOEC.

**Table 1:** Concentration–response models of individual ionic liquids and their mixtures on the inhibitory effects of luciferase luminescence and some statistics.

No.	*F*	*α*	*β*	*R* ^2^	RMSE	*f*	EC_50_	EC_10_	NOEC	*t*	*p_i_*(M1)	*p_i_*(M2)	*p_i_*(M3)
IL1	W	12.02	5.015	0.998	0.0165	0.70	3.39[3.26–3.69] × 10^−3^	1.43[1.13–1.69] × 10^−3^	7.04 × 10^−4^	1.62	2.09 × 10^−^^2^	1.82 × 10^−^^2^	1.26 × 10^−^^2^
IL2	W	6.321	6.893	0.961	0.0344	0.66	1.07[0.875–1.49] × 10^−1^	5.71[3.30–7.32] × 10^−2^	4.89 × 10^−2^	2.23	8.35 × 10^−^^1^	5.75 × 10^−^^1^	8.78 × 10^−^^1^
IL3	L	2.090	1.654	0.956	0.0381	0.70	5.45[3.26–9.66] × 10^−2^	2.56[NA–7.30] × 10^−3^	3.11 × 10^−3^	2.64	3.74 × 10^−^^2^	2.93 × 10^−^^1^	5.58 × 10^−^^2^
IL4	W	17.60	5.141	0.998	0.0202	0.70	3.21[3.00–3.45] × 10^−4^	1.38[1.02–1.65] × 10^−4^	6.55 × 10^−5^	2.46	2.02 × 10^−^^3^	1.72 × 10^−^^3^	1.18 × 10^−^^3^
IL5	L	34.97	12.11	0.994	0.0357	0.57	1.29[1.20–1.53] × 10^−3^	8.50[5.30–9.19] × 10^−4^	3.65 × 10^−4^	1.21	1.24 × 10^−^^2^	6.93 × 10^−^^3^	6.55 × 10^−^^3^
IL6	W	11.74	4.118	0.996	0.0268	0.57	1.15[1.03–1.31] × 10^−3^	4.01[2.54–5.43] × 10^−4^	8.61 × 10^−5^	1.35	5.87 × 10^−^^3^	6.18 × 10^−^^3^	1.55 × 10^−^^3^
IL7	W	14.18	5.564	0.986	0.0530	0.70	2.43[2.02–2.92] × 10^−3^	1.11[NA–1.63] × 10^−3^	4.45 × 10^−4^	1.77	1.62 × 10^−^^2^	1.31 × 10^−^^2^	7.99 × 10^−^^3^
IL8	W	13.58	5.246	0.994	0.0333	0.70	2.20[1.95–2.49] × 10^−3^	9.61[5.10–8.60] × 10^−4^	3.56 × 10^−4^	2.89	1.41 × 10^−^^2^	1.18 × 10^−^^2^	6.39 × 10^−^^3^
IL9	L	7.850	3.774	0.995	0.0096	0.70	8.32[7.70–9.05] × 10^−3^	2.18[1.82–2.53] × 10^−3^	1.14 × 10^−3^	2.85	3.19 × 10^−^^2^	4.47 × 10^−^^2^	2.05 × 10^−^^2^
IL10	W	7.272	2.692	0.995	0.0163	0.70	1.45[1.31–1.61] × 10^−3^	2.90[1.90–3.93] × 10^−4^	8.93 × 10^−5^	1.68	4.24 × 10^−^^3^	7.79 × 10^−^^3^	1.60 × 10^−^^3^
IL11	W	8.810	3.848	0.999	0.0119	0.57	4.12[2.24–5.75] × 10^−3^	1.34[1.07–1.66] × 10^−3^	4.39 × 10^−4^	1.19	1.96 × 10^−^^2^	2.21 × 10^−^^2^	7.88 × 10^−^^3^
M1	W	3.963	2.682	0.999	0.0083	0.66	2.43[2.30–2.57] × 10^−2^	4.82[3.95–5.70] × 10^−3^	1.88 × 10^−3^	2.38			
M2	W	5.112	3.251	0.981	0.0390	0.66	2.06[1.67–2.52] × 10^−2^	5.44[1.22–9.16] × 10^−3^	2.11 × 10^−3^	2.64			
M3	W	3.039	2.405	0.993	0.0181	0.66	3.83[3.37–4.38] × 10^−2^	6.31[3.65–9.17] × 10^−3^	1.93 × 10^−3^	2.53			

M1, M2, and M3 are mixtures of ILs in the ratios of their EC_50_, EC_10_, and NOEC values to their total mixture concentrations respectively, *F* is function, W is Weibull function, L is Logit function, *α* is location parameter, *β* is slope parameter, *R*^2^ is coefficient of determination, RMSE is root mean square error, *f* is geometric dilution factor, all the units of EC_50_, EC_10_, and NOEC are mol/L, EC_50_ is 50%-effect concentration, EC_10_ is 10%-effect concentration, the number in the brackets is 95% confidence interval, NA is confidence interval not available, NOEC is no observed effect concentration, *t* is Student *t*-statistic, *p_i_* is concentration (mol/L) proportion of components.

### 2.2. Mixture Toxicity

**Figure 2:**
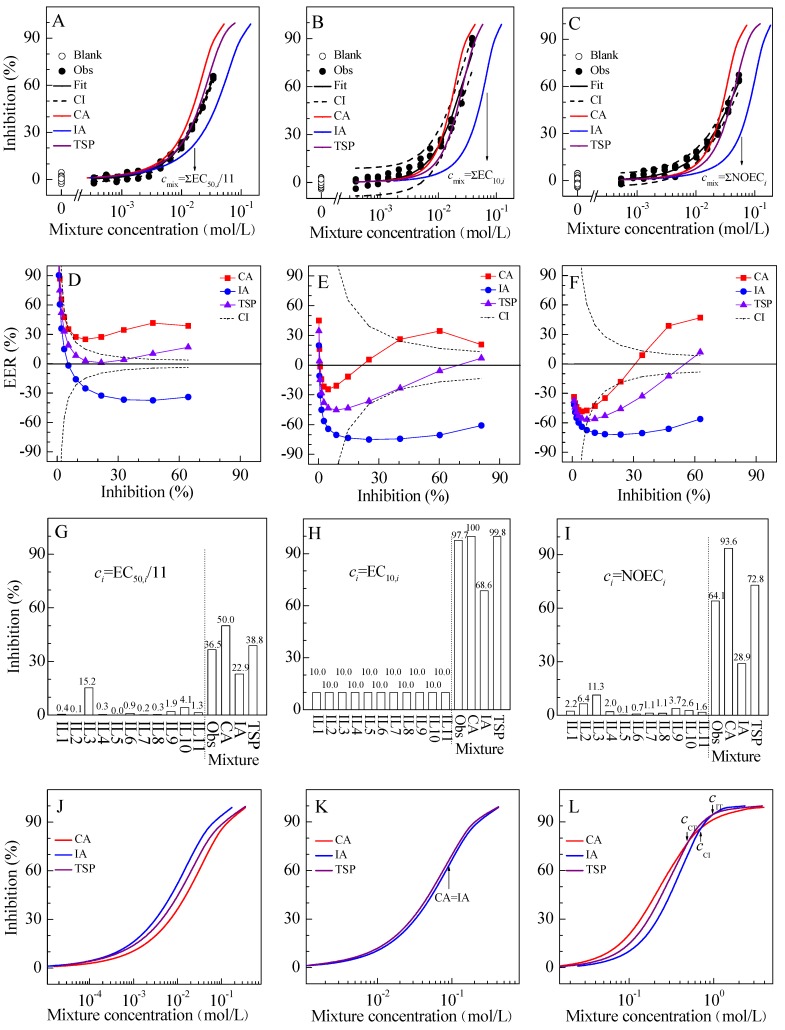
(**A**–**C**) Concentration–response curves (CRCs) for the inhibitory effects of ionic liquid (IL) mixtures of M1 (**A**), M2 (**B**), and M3 (**C**) on luciferase luminescence. (**D**–**F**) Comparison of effect residual ratio (ERR) of CA, IA, and TSP models at different effect levels for mixtures of M1 (**D**), M2 (**E**), and M3 (**F**). (**G**–**I**) Comparison of the effects on luciferase luminescence of mixtures with those of individual ILs which concentration correspond to EC_50_/11 (**G**), EC_10_ (**H**), and NOEC (**I**) respectively. (**J**–**L**) Comparison of the CRCs predicted by CA, IA, and TSP for three virtual mixtures of V1 (**J**), V2 (**K**), and V3 (**L**).

The CRCs of M1, M2 and M3 mixtures of ILs in the ratios of their EC_50_, EC_10_, and NOEC values respectively on luciferase can be effectively described by the Weibull model (Figure 2A–C). In all cases, the *R*^2^s were greater than 0.98 and the RMSEs less than 0.04. According to Table 1, it is of interest to observe a monotonically increasing relationship between the concentration ratio of IL2 and the observed mixture toxicity (EC_50_). Meanwhile, IL2 is the component having the least toxicity and the most concentration ratio in the mixtures. Figure 2D–F show the comparison of effect residual ratio (ERR) of the TSP, CA, and IA models at different effect levels for M1, M2 and M3 mixtures. The ERR of 95% confidence interval (CI) for each mixture was also given. It is possible to compare the TSP, CA, and IA models’ evaluations of mixture toxicity. As a result, the toxicities of M1 can be effectively predicted by TSP, but were overestimated by CA and underestimated by IA. The toxicities of M2 can be basically predicted by TSP and CA, but were underestimated by IA. The toxicities of M3 can be predicted by TSP and CA in a certain concentration range, but were underestimated by IA. As the exposure concentration increased, the CRCs of these mixtures deviated from CA and approached IA, which could indicate that the mechanisms of action (MoAs) of ILs in the mixtures can shift with dose. For M1–M3 mixtures, CA predicted a higher effect than IA. The same situation can be found in other studies [2,3]. For this case, CA is more conservative than IA from the viewpoint of risk assessment. CA has been proved to be a more generalized approach for the effect prediction of mixtures, especially with non-monotonic CRCs [23] and with effects unable to normalize to 100% [30,31]. For mixtures consisting of different types of chemicals and deviating from additive models, we suggested that besides CA and IA, TSP should be used side by side if possible.

To use the TSP method effectively, it should be ensured that the concentrations and CRC models of mixture components are known, the inverse functions of component CRC models are available, and the effects can be normalized to 0–100% range. Unfortunately, the MoAs of chemicals are unknown in most cases, and sometimes one chemical may have several MoAs [32]. So it is unrealistic to determine the specific MoAs for various components. There may be certain degree of subjectivity in the TSP grouping. In this case, it is necessary to select at least one mixture for verifying the effectiveness of the grouping scheme. It has been demonstrated that the TSP according to molecular similarity grouping gives accurate prediction for the effects of M1–M3 mixtures in our study. Additionally, in the absence of knowledge on the MoAs of the components, a quantitative structure–activity relationship (QSAR) based TSP model based on structural similarity analysis might be applied to predict mixture toxicity effectively [33].

Focusing on the selected points (marked by arrows in Figure 2A–C) of the CRCs of M1, M2 and M3 mixtures, the mixtures of ILs mixed at low concentrations were found to produce significant effects (Figure 2G–I). These effects of mixtures were more effectively predicted by TSP, but were overestimated by CA and underestimated by IA. In Figure 2I, the mixture of ILs mixed at NOEC could cause significant effect of 64.1% that was nearly twice the sum (33.0%) of component effects. Similar results can be found in the report [34]. This result emphasized the unsuitability of NOEC as an approximation of no effect concentration (NEC) especially in the context of mixtures. In Figure 2H, the mixture of ILs mixed at EC_10_ could cause a significant effect of 97.7%. This result indicated that EC_10_ as an alternative to NOEC was not necessarily conservative in the case of combined exposures. In Figure 2G, the mixture of ILs mixed at 1/11 of individual EC_50_ produced 36.5% effect. For the assessment factors in U.S. Food Quality Protection Act of 1996 [35], a 10-fold reduction in exposure was accounted for differences between humans and animals, a further 10-fold reduction was accounted for human-to-human variability, and another 10-fold reduction was added for protecting infants and children. In the absence of low-effect concentrations such as NOEC and EC_10_, the EC_50_ divided by the assessment factors may be considered for evaluating low-concentration and combined exposures. Additionally, CA predicted an effect of 50% in Figure 2G, which proved the hypothesis that a mixture of *n*-component mixed at concentrations of *EC_x_*_,*i*_/*n* will theoretically cause an effect of *x*% based on CA [36].

### 2.3. Location Relationship among CA, IA, and TSP Curves

Mathematical proof indicates that CA-predicted effect will be greater than, equal to, or less than IA-predicted effect when Weibull *β* values of all components are greater than, equal to, or less than 2.3 (*i.e.*, ln10) [37]. In the present study, CA curves are all above IA curves for M1–M3 mixtures (*i.e.*, CA > IA), which can be attributed to Weibull *β* values of the ILs being greater than 2.3 except for that of IL3 being 1.34. In this case, TSP curves are always located between the CA and IA curves. Similar results can be found in other studies [4,5].

For other cases, we designed three virtual mixtures in EC_50_ ratios named as V1, V2, and V3. V1 included the four components (C1–C4) with Weibull parameters (*α*, *β*) as C1 (2.0, 1.8), C2 (2.6, 1.4) in one group, and C3 (2.3, 1.6), C4 (2.9, 1.2) in the other group. V2 included the four components (C5–C8) with Weibull parameters (*α*, *β*) as C5 (2.0, 2.3), C6 (2.6, 2.3) in one group, and C7 (2.3, 2.3), C8 (2.9, 2.3) in the other group. V3 included the four components (C9–C12) with Logit parameters (*α*, *β*) as C9 (2.0, 3.8), C10 (2.6, 3.8) in one group, and C11 (2.3, 3.8), C12 (2.9, 3.8) in the other group. For V1, the order of mixture effects was IA > TSP > CA, and TSP curve was located between the CA and IA curves (Figure 2J). Interestingly, the order of mixture effects was TSP > CA = IA for V2, and TSP curve was above CA and IA curves (Figure 2K). For V3, there are three intersections between CA, IA and TSP curves at the concentrations of *c*_CT_ (CA and TSP intersecting concentration), *c*_CI_ (CA and IA intersecting concentration), and *c*_IT_ (IA and TSP intersecting concentration) with the order of *c*_CT_ < *c*_CI_ < *c*_IT_; TSP curve was located between the CA and IA curves in the range of concentration smaller than *c*_CT_ and greater than *c*_IT_; TSP curve was above CA and IA curves in the range of concentration greater than *c*_CT_ and smaller than *c*_IT_ (Figure 2L). Based on the results of experimental verification and numerical simulation mentioned above, we proposed a hypothesis that TSP curve will be located between the CA and IA curves when there is no overlapping or intersecting between CA and IA curves (*i.e.*, CA ≠ IA). This hypothesis is worthy of further experimental investigation and mathematical proof.

## 3. Experimental Section

### 3.1. Chemicals

**Figure 3:**
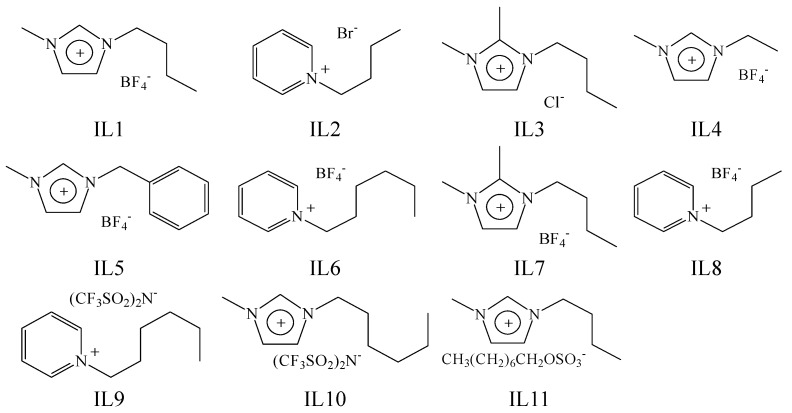
Chemical structure of the test ionic liquids.

The IL components included seven compounds from Acros (Geel, Belgium), namely 1-butyl-3-methylimidazolium tetrafluoroborate (IL1, purity 99.2%, CAS 174501-65-6), 1-butylpyridinium bromide (IL2, purity 98%, CAS 874-80-6), 1-butyl-2,3-dimethylimidazolium chloride (IL3, purity 99.3%, CAS 98892-75-2), 1-ethyl-3-methylimidazolium tetrafluoroborate (IL4, purity 99%, CAS 143314-16-3), 1-benzyl-3-methylimidazolium tetrafluoroborate (IL5, purity 97%, CAS 500996-04-3), 1-hexylpyridinium tetrafluoroborate (IL6, purity 97.5%, CAS 474368-70-2), and 1-butyl-2,3-dimethyl-imidazolium tetrafluoroborate (IL7, purity 99%, CAS 402846-78-0); three from Merck (Darmstadt, Germany), 1-butylpyridinium tetrafluoroborate (IL8, purity 99.2%, CAS 203389-28-0), 1-hexyl-pyridinium bis(trifluoromethylsulfonyl)imide (IL9, purity 98%, CAS 460983-97-5) and 1-hexyl-3-methylimidazolium bis(trifluoromethylsulfonyl)imide (IL10, purity ≥ 98%, CAS 382150-50-7); and one from Strem (Newburyport, MA, USA), 1-butyl-3-methylimidazolium octylsulfate (IL11, purity 98%, CAS 445473-58-5). The chemical structures of these ILs are shown in Figure 3. 

The stock solutions of these IL were prepared by dissolving them in Milli-Q water and they were stored in the dark at 4 °C. The stock solutions of IL mixtures were prepared by mixing the stock solutions of individual ILs according to their assigned concentration ratios. The chemicals used in the luciferase luminescence include adenosine-5'-triphosphate (ATP-Na_2_, ≥ 98.0% purity) from Sigma-Aldrich (St. Louis, MO, USA), the QuantiLum recombinant luciferase (cloned from North American firefly *Photinus pyralis*, Catalog #E1701, > 95% purity) and endotoxin-free D-luciferin (Catalog #E6551, ≥ 98.5% purity) from Promega (Madison, WI, USA), and the glycylglycine buffer (pH 7.8, consisting of 50 mmol/L glycylglycine, 1 mmol/L MgSO_4_, 0.5 mmol/L EDTA, and 10 mmol/L DTT) [38]. Luciferase, luciferin, and ATP were separately stored in the glycylglycine buffer.

### 3.2. Luciferase Toxicity Test

The toxicities of single ILs and their mixtures were expressed as percentage inhibition of the cell‑free luciferase luminescence system. According to the microplate toxicity analysis (MTA) developed in our previous study [39], the relative light units (RLUs) of the luciferase luminescence exposed to single ILs and their mixtures were determined on SpectraMax M5 reader (Molecular Devices Inc., San Francisco, CA, USA) with a 96-well white flat bottom microplate. IL chemicals and their mixtures with 12 concentration series in triplicates and 12 controls were arranged in a microplate as follows: 100 µL water was added to 12 wells in the first row as blank controls, 100 µL of the solutions of IL chemicals and their mixtures with 12 gradient concentrations according to a geometric dilution factors (*f*) were added to 12 column wells from the second to the fourth row. Then, 50 µL ATP of 5.5 × 10^−4^ mol/L, 50 µL luciferin of 6.5 × 10^−5^ mol/L and 50 µL luciferase of 3.3 × 10^−7^ mol/L were added into each test well to reach the final test volume of 250 µL [40]. The test of each chemical and mixture was repeated in three microplates.

The RLUs in the microplate wells were determined after 15 min of exposure at 25 °C. The effect (*E* of *x*%) of individual IL chemicals and their mixtures was calculated as Equation (1). The CRCs were fitted by Logit (L) or Weibull (W) function [3] using least squares method. The analytical formulas for the function (*F*) and the inverse function (*F*^−1^) of Logit were given as Equations (2) and (3), and that of Weibull were given as Equations (4) and (5). The goodness of fit of statistical models was evaluated by coefficient of determination (*R*^2^) and root mean square error (RMSE). As a quantitative measure of the uncertainty, the observation-based 95% confidence interval (CI) was determined [41].

*E* = *x*% = (1 − *L/L*_0_) × 100% (1)

*E* = 1/(1 + exp(− *α* − *β* × log_10_(*c*))) (2)

*c* = 10^((1n(*E*/(1−*E*)− *α*/*β*)^ (3)

*E* = 1 − exp(−exp(*α* + *β* × log_10_(*c*))) (4)

*c* = 10^((1n(−1n(1−*E*)) − *α*)/β^ (5)

where *L*_0_ is an average of RLUs of 12 controls, *L* is an average of RLUs of 3 treatments, *E* is the effect, *c* is IL concentration, *α* is location parameter, and *β* is slope parameter.

To conduct the low-concentration assessment, the no observed effect concentration (NOEC) of chemicals were determined by using Dunnett test [42]. NOEC is the highest test concentration that does not statistically significantly deviate from the control. To test the significance of this difference between treatments and control, a Student *t*-statistic was calculated according to one control in triplicates (three microplates) and 12 treatments in triplicates (three microplates). In our study, the critical value of *t* was 2.98 for the 0.05 significance level (*a*), 26 degrees of freedom (*df*), and 12 treatments (*p*). Then, NOEC is the highest test concentration with *t* value less than 2.98.

### 3.3. Experimental Design and Toxicity Prediction of Mixtures

We designed three IL mixtures named as M1, M2, and M3, where various ILs were in the ratios of their individual EC_50,*i*_, EC_10,*i*_, and NOEC*_i_* to the total concentration (∑EC_50,*i*_, ∑EC_10,*i*_, and ∑NOEC*_i_*) of the mixtures, respectively. The mixture effects were predicted by models of CA [Equation (6)], IA [Equation (7)] [3], and TSP [4]. For TSP, since the specific MoAs of the 11 ILs on luciferase were unknown, we assigned the seven imidazolium ILs into one group and the four pyridinium ILs into the other group according to their molecular similarity. Then, the mixture effect of ILs in the same group was predicted by CA using Equation (6) in the first stage, and the overall mixture effect of the two group ILs was predicted by IA using Equation (7) in the second stage.

ERR method had been demonstrated to be an effective way for comparing the deviation of CA and IA from observation [43]. The ERR [Equation (8)] was defined as a ratio of the difference between the effect (*E*) predicted by a reference model at a certain concentration and that observed to the observed effect. To effectively evaluate the predictability of the CA, IA, and TSP models in our study, ERR was used for the quantitative comparison of model prediction errors at different effect levels:

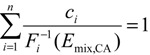
(6)

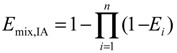
(7)

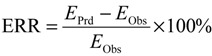
(8)
where *n* is the number of mixture components, *c_i_* is the concentration of the *i*th component in the mixture, *F*_*i*_^−1^ is the inverse of function describing the CRC of the *i*th component, *E*_mix,CA_ is mixture effect predicted by CA, *E*_mix,IA_ is mixture effect predicted by IA, *E_i_* is the effect of the *i*th component in a mixture, *E*_Prd_ is the effect predicted by a reference model, and *E*_Obs_ is the effect observed.

## 4. Conclusions

We have studied the combined effects of seven imidazolium ILs and four pyridinium ILs on luciferase. Overall, the combined effects can be more effectively predicted by the two-stage prediction (TSP) method than the concentration addition and independent action models. This supports the use of the TSP method as a default approach for predicting the combined effect of different types of chemicals. It was demonstrated that even low concentrations (NOEC or EC_10_) of chemicals may lead to a significant overall effect when acting simultaneously. Therefore, the combined effects of pollutant mixtures should be taken into account in environmental risk assessment.
